# High Concentrate Diet Induced Mucosal Injuries by Enhancing Epithelial Apoptosis and Inflammatory Response in the Hindgut of Goats

**DOI:** 10.1371/journal.pone.0111596

**Published:** 2014-10-30

**Authors:** Shiyu Tao, Yongqian Duanmu, Haibo Dong, Yingdong Ni, Jie Chen, Xiangzhen Shen, Ruqian Zhao

**Affiliations:** 1 Key Laboratory of Animal Physiology & Biochemistry, Ministry of Agriculture, Nanjing Agricultural University, Nanjing, Jiangsu, China; 2 Institute of Small Animal Disease, College of Veterinary Medicine, Nanjing Agricultural University, Nanjing, Jiangsu, China; USGS National Wildlife Health Center, United States of America

## Abstract

**Purpose:**

It is widely accepted that lipopolysaccharide and volatile fatty acids (VFA) accumulate in the digestive tract of ruminants fed diets containing high portions of grain. Compared to the ruminal epithelium, the hindgut epithelium is composed of a monolayer structure that is more “leaky” for lipopolysaccharide and susceptible to organic acid-induced damage. The aim of this study was to investigate changes in epithelial structure, apoptosis and inflammatory response in the hindgut of goats fed a high-concentrate diet for 6 weeks.

**Experimental Design:**

Eight local Chinese goats with rumen cannulas were randomly assigned to two groups: one group was fed a high-concentrate diet (65% concentrate of dry matter, HC) and the other group was fed a low-concentrate diet (35% concentrate of dry matter, LC) for 6 wks. Ruminal fluid, plasma, and hindgut mucosa tissues were collected. Histological techniques, real-time PCR and western blotting were used to evaluate the tissues structure, cell apoptosis and local inflammation in the hindguts.

**Results:**

Feeding HC diet for 6 wks resulted in a significant decrease of ruminal pH (p<0.01), and ruminal lipopolysaccharide concentrations were significantly increased in HC goats (p<0.05). Obvious damage was observed to mucosal epithelium of the hindgut and the intercellular tight junctions in HC, but not in LC, goats. The expression of MyD88 and caspase-8 mRNA was increased in colonic epithelium of HC goats compared to LC (p<0.05), and the expression of TLR-4 and caspase-3 showed a tendency to increase. In the cecum, interleukin-1β mRNA expression was decreased (p<0.05), and caspase-3 showed a potential increase (p = 0.07) in HC goats. The level of NF-κB protein was increased in colonic epithelium of HC goats. Caspase-3 activity was elevated in both colon and cecum, whereas caspase-8 activity was statistically increased only in colon.

**Conclusions:**

Feeding a high-concentrate diet to goats for 6 wks led to hindgut mucosal injuries via activating epithelial cells apoptosis and local inflammatory response.

## Introduction

Current intensive production systems for ruminants feed large amounts of grain to animals to maximize energy intake and support high milk yields or rapid growth. These feeding programs might be useful to lower the cost in the short term, however, the excessive amounts of non-structural carbohydrates and highly fermentable forages result in rapid fermentation and organic acids accumulation in rumen and hindgut [1], [2]. Moreover, feeding excessive grain to ruminants likely leads to the accumulation of bacterial endotoxin (lipopolysaccharide, LPS) derived from gram-negative bacteria in the gastrointestinal (GI) tract [3], [4]. The increased LPS in the GI tract, especially in the hindgut, can increase the permeability for LPS [5], leading to the translocation of LPS from GI tract into circulatory system. Therefore, the accumulation and translocation of LPS might cause damages to the histological structure of mucosa barrier in GI tract [6].

Under normal conditions, the rumen epithelium prevents the translocation of toxic compounds into blood depending on the multi-cellular structure [7]. Recent findings suggest that the combination of low pH and high LPS concentration in the rumen may not cause the translocation of LPS [8]. However, there was a high risk of LPS translocation from the large intestine due to the monolayer structure when ruminants suffered a grain-induced subacute ruminal acidosis (SARA) challenge [9]. Tight junctions (TJs) play an important role in maintaining the barrier function of epithelial cells, preventing the translocation of LPS and other toxic compounds from the intestinal tract into the circulatory system [10]. The impairment of the epithelial TJ barrier can result in intestinal inflammation, including necrotizing enterocolitis, celiac disease, and Crohn's disease [11]–[13]. It's reported that the disruption of intestinal TJs can increase the permeability of intestinal epithelia, the translocation of LPS and even cause systemic inflammation [14].

Intestinal epithelial cells contribute to the regulation of inflammatory conditions and create an intestinal barrier against invading pathogens [15]. Toll-like receptors (TLRs) in the gut epithelium play a key role in maintaining the homeostasis by recognizing ligands known as microbial-associated molecular patterns (MAMPs) derived from both pathogenic and non-pathogenic bacteria [16]–[18]. After combining with TLR-4 on the host cell surface, LPS activates myeloid differentiating factor 88 (MyD88), and then elicits a pro-inflammatory NF-κB-dependent signaling cascade [19], [20]. As a major transcription factor and a first responder to harmful cellular stimuli, NF-κB plays a central role in inflammation through its ability to induce transcription of proinflammatory genes [21]. Systemic LPS administration caused a rapid intestinal epithelial cells apoptosis and the activation of caspases, paralleled a significant disruption in the intestinal villi and the epithelial cells shedding [22].

Several studies conducted in goat and dairy cattle showed that a high grain diet impaired barrier functions of the rumen epithelium [23], [24], inflammation and caused other health problems in host animals [25]. Several chaperone proteins with important cellular protective functions in epithelial tissues were collectively down-regulated in response to high dietary energy supply, which indicates that energy-rich diets and the resulting acidotic insult may facilitate the impairment of rumen barrier function by attenuating their cell defense system [26]. However, no information exists about the underlying mechanism behind the disruption of protective functions of the hindgut epithelium in response to high grain diet to ruminants. Based on the finding that the rumen digesta and presence of LPS make the hindgut tissues “leaky” [27], we hypothesized that feeding goats a diet enriched with a high level of concentrate could cause structure damages in hindgut epithelium via triggering epithelial cells apoptosis as well as the local inflammatory response. Therefore, the aim of this study was to investigate the changes of epithelial structure, apoptosis and inflammatory response in the hindgut of goats after feeding a high-concentrate diet for 6 wks.

## Results

### Ruminal pH

**Figure 1:**
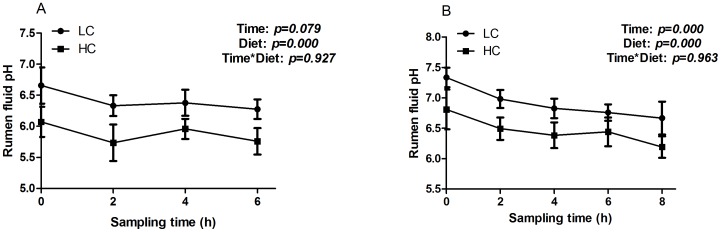
pH value in ruminal fluid after 2 (A) and 6 weeks (B) feeding regime. Data was analyzed for differences due to diet, time, and their interactions by ANOVA using the General Linear Models of SPSS 11.0 for Windows (StatSoft Inc, Tulsa, OK, USA). Data was considered statistically significant when P<0.05, n = 4.

After feeding a high concentrate diet for 2 and 6 wks, ruminal pH was significantly decreased in HC goats compared to control (*p<0.01*). Ruminal pH was affected significantly by digesting time (*p<0.01*), while there was no interaction of digesting time and diet on ruminal pH (*p>0.05*; *figure 1A–B*).

### Concentrations of LPS in rumen and plasma

**Figure 2:**
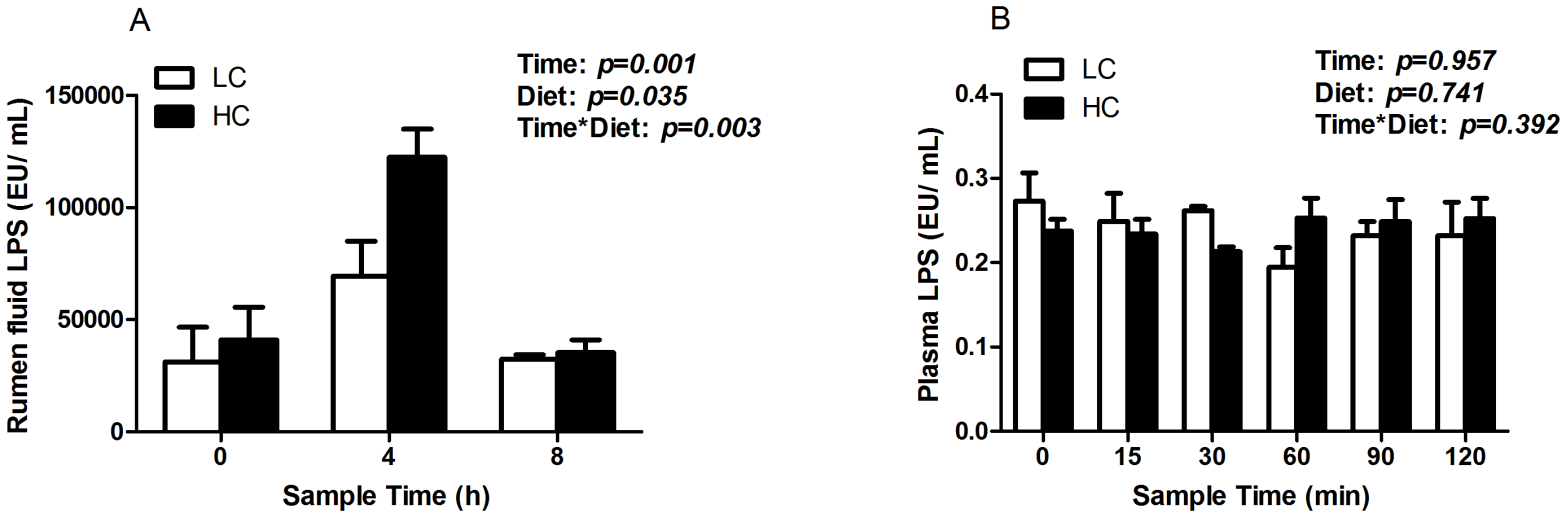
LPS concentrations in ruminal fluid (A) and plasma (B). The results were expressed as mean ± SEM. Data were analyzed for differences due to diet, time, and their interactions by ANOVA using the General Linear Models of SPSS 11.0 for Windows (StatSoft Inc, Tulsa, OK, USA). Data was considered statistically significant when P<0.05, n = 4. * P<0.05 between two groups.

As shown in Figure 2A, both digesting time (*p<0.01*) and diet (*p<0.05*) significantly affected LPS content in rumen fluids, and with a significant higher level of LPS in ruminal fluids of HC goats compared to control. There was a significant interaction between time and diet on LPS content in rumen fluids (*p<0.01*; *figure 2A*). However, the level of LPS in blood did not show significant difference between HC and LC goats (*p>0.05;* *figure 2B*).

### Morphology of TJs and mucosa tissue in the hindgut

**Figure 3:**
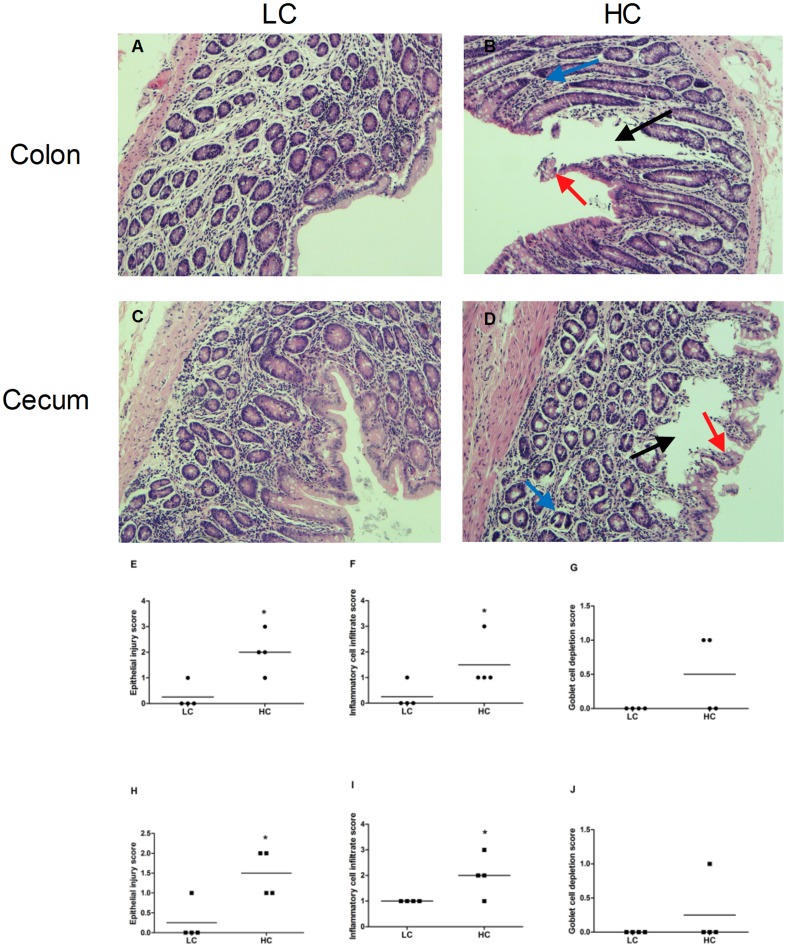
Comparisons of the hindgut (colon and cecum) histology between HC and LC goats. Hindgut (n = 4) from each group were processed for histological evaluation: colon section of the (A) LC group; (B) HC group and cecum section of the (C) LC group; (D) HC group. The representative histological sections of the hindgut were stained by H&E at 400× magnification. Black, blue and red arrow indicates the loss of epithelial integrity, inflammatory cell infiltrate and goblet cell depletion of the hindgut epithelium, respectively. E–J: Histological damage score in colon (E–G) and cecum (H–J) mucosa. Asterisks denote a significant difference compared with control.

Hematoxylin and eosin (H&E) staining showed that in colon and cecum epithelium, desquamation and severe cellular damage was observed in HC goats, whereas LC goats exhibited structural integrity of the epithelial cell morphology (*Figure 3A–D*). Compared to LC goats, HC goats showed significantly higher epithelial injury score and inflammatory cell infiltration score in both colon and cecum mucosa (*p<0.01;* *figure 3E–J*).

**Figure 4:**
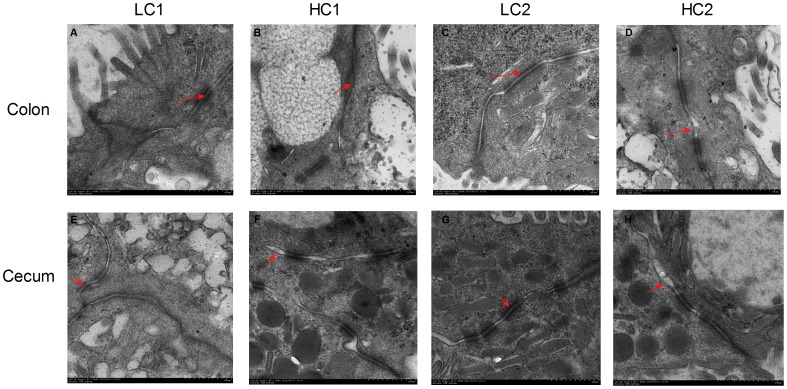
Comparisons of morphology of the hindgut mucosa (colon and cecum) between HC and LC goats. Hindgut (n = 4) from each group were processed for morphological evaluation: colon section of the (A, C) LC group; (B, D) HC group and cecum section of the (E, G) LC group; (F, H) HC group (transmission electron microscopy, ×10000). Arrow indicates the location of the TJs (Scale bar = 500 nm).

The ultrastructure of TJ in the hindgut was detected by transmission electron microscopy (TEM) to determine the hindgut TJ status. As shown in Figure 4A–H, compared to control, the TJs were damaged and the intercellular space between epithelial cells widened in HC goats (*Figure 4A–H*).

### Gene expression in hindgut mucosa tissues

**Figure 5:**
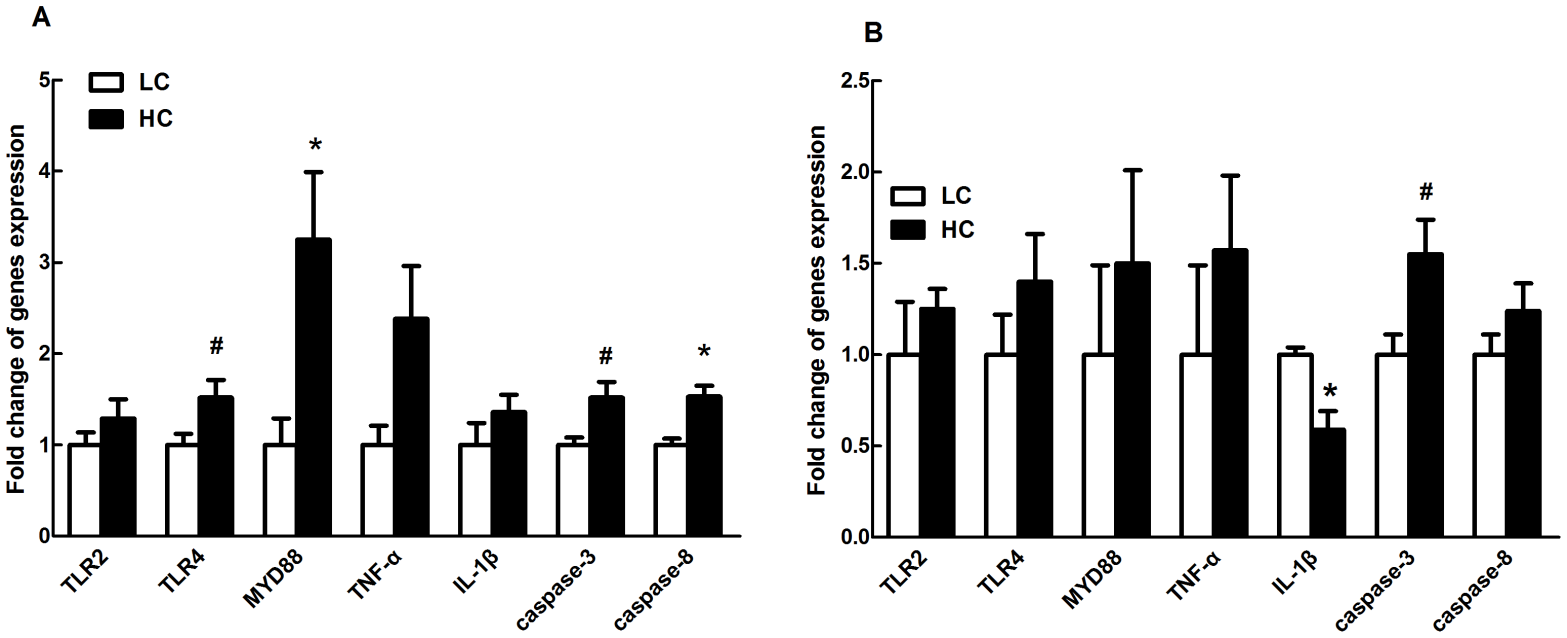
Gene expression in colon (A) and cecum mucosa (B). Beta-actin was used as the reference gene for gene expression. The data were analyzed by Independent-Samples T test using the Compare Means of SPASS 11.0 for Windows (StaSoft Inc, Tulsa, OK, USA). Values are mean ± SEM. *^#^p<0.1*, *p<0.05 vs. LC.

In colon mucosa, MyD88 and caspase-8 mRNA expressions were significantly increased (*p<0.01*), and there was a trend toward increased mRNA expression of TLR-4 (*p = 0.07*) and caspase-3 (*p = 0.06*) in HC goats compared to control (*Figure 5A*).

In cecum mucosa, IL-1β mRNA expression was significantly decreased in HC goats compared to control counterparts (*p<0.05*), and there was a potential increase of caspase-3 mRNA expression in HC goats (*p = 0.07*; *figure 5B*).

### Caspase activity in the hindgut mucosa tissues

**Figure 6:**
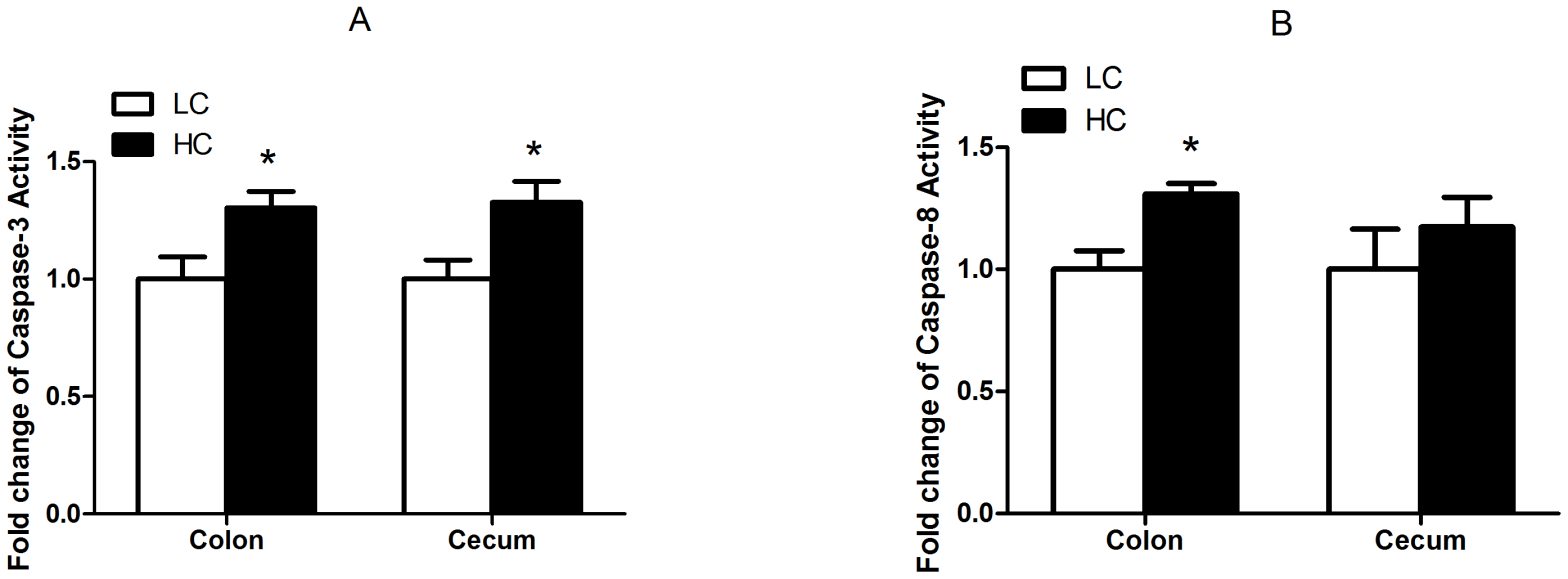
Caspase-3 (A) and caspase-8 (B) enzyme activity in the hindgut mucosa. The results were expressed as mean± SEM. The data were analyzed by Independent-Samples T test using the Compare Means of SPASS 11.0 for Windows (StaSoft Inc, Tulsa, OK, USA). *p<0.05 vs. LC.

As shown in Figure 6A, HC goats showed markedly higher level of caspase-3 enzyme activity in colon and cecum than that in LC goats (*p<0.05*), and higher caspase-8 activity in colon mucosa (*p<0.05*) but not in cecum mucosa (*p>0.05*; *figure 6B*).

### NF-kB protein content in the hindgut tissues

**Figure 7:**
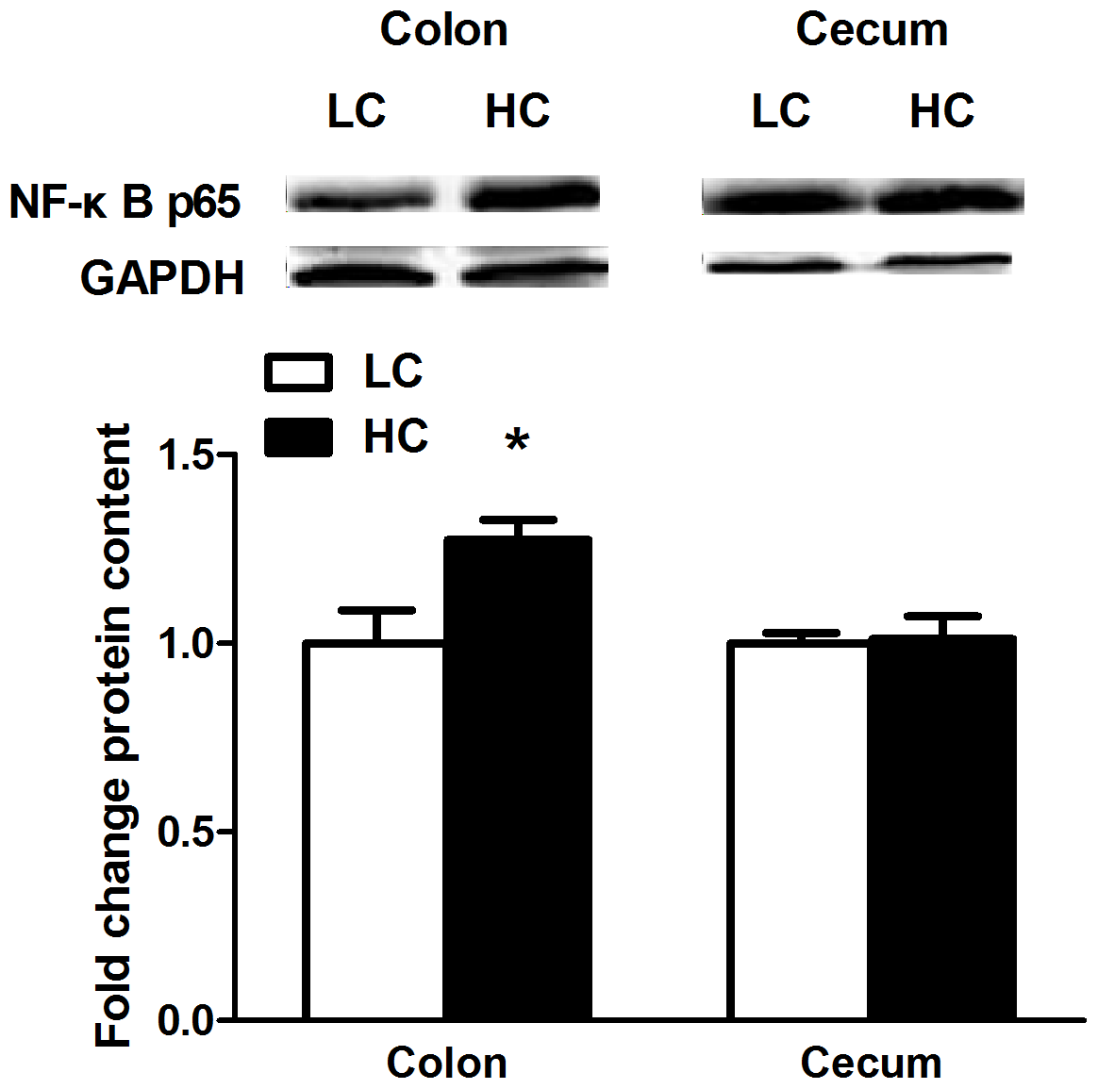
Protein expression of NF-κB p65 in colon and cecum mucosa. Results of NF-κB p65 protein levels expressed as arbitrary units relative to β-actin protein. Values are mean ± SEM. The data were analyzed by Independent-Samples T test using the Compare Means of SPASS 11.0 for Windows (StaSoft Inc, Tulsa, OK, USA). *^#^p<0.1*, *p<0.05 vs. LC.

Compared to LC goats, HC goats demonstrated a significant increase of NF-kB protein in colon mucosa (*p<0.05*), while there was no significant difference in cecum mucosa (*p>0.05*; *figure 7*).

## Discussion

In ruminants, SARA is a metabolic disease induced by feeding excessive amounts of highly fermentable forages and insufficient dietary coarse fiber [28], [29]. Several rumen pH thresholds (e.g., 6.0, 5.8, 5.6, and 5.5) have been used to define SARA [30] [31], [32]. In the present study, ruminal pH in HC goats fed a diet with 65% concentrate decreased to less than 5.8 at 2 h after feeding and continued for approximately four hours during wk 2. However, after feeding 6 wks, although ruminal pH of HC goats was still significantly lower than the control goats, the value was increased to more than 6.0, indicating the adaptation to high concentrate diets after a long-term feeding. This adaptation phenomenon was also observed in Steele's experiment [6]. Similarly, feeding 60% grain diet to growing goats for 6 wks induced a lower ruminal and colonic pH, and also altered genes expression associated with nutrient transport and electrochemical gradients in rumen and colon epithelium, indicating an increase systemic absorption of VFA to counteract the consequences of luminal acidification on intracellular homeostasis in epithelial cells [33].

Previous studies suggested that ruminants fed diets enrich with starch resulted in the increase of rumen LPS concentration [34], [35]. In a good agreement, our results showed that LPS content in rumen fluids of HC goats was markedly higher than the control goats. Li *et al* also found that a grain-based SARA in dairy cow led to a huge increase of LPS concentration in cecal digesta and wet feces [9]. In growing goats, colonic LPS concentration significantly increased in response to the 60% grain diet for 6 wks, whereas its ruminal concentration decreased in response to the same diet [36]. Unfortunately, the concentration of LPS in hindgut digesta was not recorded in this study due to the insufficient amount of samples. As in the previous report [9], our results showed that all concentrations of LPS in blood plasma were below 0.04 EU/mL, and there was no significant difference between HC and LC goats. The grain-based SARA challenge caused translocation of LPS from the digestive tract but that LPS was detoxified before entering the peripheral blood circulation [9]. It is well known that acidic conditions in the abomasums lead to degradation of LPS [6] and the Kupfer cells in liver have high clearance rate of the LPS [37]. However, feeding 65% grain diets to the male goats for 7 weeks resulted in translocation of LPS into the blood with the concentrations of 0.8±0.20 EU/mL, suggesting a leakage of LPS into the blood [23]. Klevenhusen *et al* found that in growing goats, greater amounts of grain in the diet were associated with a quadratic increase in serum LPS concentration compared with controls (0.04 vs 0.08 EU/mL), whereas no signs of inflammation became apparent, as serum amyloid A (SAA) concentrations remained unaffected by diet [1]. Taken together, these results suggest that increased LPS release in the rumen or hindgut during lyses of gram-negative bacteria exposed to acidic conditions might have contributed to epithelial damage [9], [38].

The cellular structure of the ruminal epithelium is complex, consisting of a multilayered structure with intercellular tight junctions (TJs) in the two middle layers. However, hindgut epithelium only has a monolayer structure [38]. Histological differences between the rumen and the hindgut suggest that the barrier function of the hindgut epithelium is more easily compromised by high level of LPS [9]. Effects of high grain diet on ruminal defense function and epithelial permeability have been well documented [24], [27]. In the present study, obvious desquamation and severe cellular damage were observed in colon and cecum epithelium of HC goats, while all LC goats maintained normal and healthy epithelial structure. Our results also showed that the intercellular space was obviously widened in colon and cecum epithelium of HC goats, indicating the disruption and expansion of TJs in the hindgut tissues. It is reported that the TJs opening could regulate the paracellular pathway, and it might be related to disrupted permeability of digestive tract [39], [40]. In this study, as previously reported [41], the mechanical widening of the TJs (*Figure 4*) indicates the disruption of TJ proteins and the loss of TJ integrity. Several factors such as pH value and toxic compounds have been suggested to involve in the process of structure disruption in epithelial tissues caused by feeding high grain diet [9], [42]. The level of organic acids in colonic digesta was significantly increased in dairy cattle fed high grain diet, therefore, resulting in acidotic insult in the hindgut [9]. In a human colon adenocarcinoma cell line, acetate treatment in the pH range of 6.0 to 7.0 induced cell apoptosis rather than necrosis, while acetate treatment at pH 5.5 caused cell necrosis [7]. Additionally, a recent study conducted in growing goats showed that after feeding high grain diet for 6 wks, several chaperone proteins with important cellular protective functions were collectively down-regulated in ruminal epithelium, indicating an attenuated cell defense system [26].

Feeding high grain diet to growing goats resulted in enhanced concentration of LPS in colonic digesta [38]. Increased release of LPS in colonic lumen exposed to acidic conditions might have contributed to epithelial damage by its ability to trigger inflammatory response. As one of the most potent inflammatory mediators and a major structural component of Gram-negative bacteria, LPS has been hypothesized to form an important risk factor of inflammatory bowel disease (IBD) [43]. LPS activates the host Toll-like receptor 4 (TLR4) to induce the production of pro-inflammatory cytokines and apoptosis of intestinal epithelial cells in IBD, which contribute to cytokine-mediated mucosal tissue damage, leading to a breakdown in the mucosal barrier [44]. MyD88-dependent pathway is the downstream signals of TLR-4, and initiation of MyD88-dependent pathway could lead to activation of NF-κB and transcription of several pro-inflammatory genes [16]. Diverse stimuli (e.g., microbial products, microbes, pro-inflammatory cytokines, and oxidative stress) can activate NF-κB, and the downstream cytokines have been used to assess inflammation [3], [23]. In this study, the mRNA transcript level of TLR-4 in colon mucosa was up-regulated in HC goats, and MyD88 mRNA expression showed a significant up-regulation with a 3-fold increase compared to control goats. TNF-α mRNA expression in colon epithelium of HC goats was increased by 1.5 fold compared to control. Moreover, the level of NF-κB p65 protein was significantly enhanced in colonic mucosa of HC goats. NF-κB activation has been detected in the mucosa of patients with IBD and in murine colitis model, and inhibition of NF-κB with a specific p65 antisense oligonucleotide is effective in preventing experimental models of IBD and efficiently down-regulates cytokine production [45]. Therefore, the activation of TLR-4 signaling pathway in colonic epithelium indicates the presence of an inflammatory response in HC goats after 6 wks feeding high concentrate diets. However, in cecum epithelium, except for a significant decrease of IL-1β mRNA expression there was no difference of TLR4, MyD88 mRNA expression, or NF-κB p65 protein translation between HC and control goats, suggesting other factors or pathways (e.g., apoptosis pathway) contributed to the epithelial damage.

Apoptosis was associated with activated caspase-3, -8, -9 and -10, and inactivation of caspase 10 or 3 was sufficient to block apoptosis in this pathway [46]. In epithelial tissues, tight junction damage or disruption is usually thought of as a downstream consequence of caspase cleavage during the apoptotic process [47]. In another study, signaling through tumor necrosis factor (TNF) leads to the recruitment of signaling complex that activates caspase-8, which then mediates apoptosis by activating caspases including caspase-3, suggesting the important roles of caspase-3 and -8 in mediating cell apoptosis and tissues damage. Our results showed that both mRNA transcription and the activities of caspase-3 and -8 were markedly enhanced in colon epithelium of HC goats compared to control. In cecum epithelium, the transcript level of caspase 3 showed a tendency to increase, and its activity was significantly enhanced in HC goats. It is reported that a high concentration of LPS increased localization epithelial apoptosis and permeability and these changes were dependent on caspase-3 activation [5]. Taken together, in the present study, the activation of caspase activity may contribute to the structural damage in colon and cecum epithelium of goats after feeding 6 wks of high concentrate diets.

## Conclusions

We report herein for the first time that ruminal pH dropped to the threshold for defining SARA after feeding HC diet to goats for 2 wks. After 6 wks, although ruminal pH of HC goats was lower than that of LC goats, which was increased to above 6.0 due to the adaptation to high grain diet. LPS concentrations in ruminal fluids but not in plasma were significantly increased in HC goats compared to LC. Obvious structural disruption was observed in colon and cecum mucosa barrier in HC goats but not in LC counterparts. Activations of both cell apoptosis and inflammatory response contributed to the process of epithelial damage in colon mucosa, while in cecum mucosa the activation of cell apoptosis was the dominant mechanism responsible for epithelial disruption in HC goats.

## Materials and Methods

### Ethics Statement

All animal procedures were approved by the Institutional Animal Care and Use Committee (IACUC) of Nanjing Agricultural University. The protocol of this study was reviewed and approved specifically, with the project number 2011CB100802. The slaughter and sampling procedures strictly followed the “Guidelines on Ethical Treatment of Experimental Animals” (2006) No. 398 set by the Ministry of Science and Technology, China and the Regulation regarding the Management and Treatment of Experimental Animals” (2008) No. 45 set by the Jiangsu Provincial People's Government.

### Animals and experimental procedures

Eight 6-month-old Chinese boer goats with approximately 22.0±2.0 kg body weight were housed in individual stalls in a standard animal feeding house at Nanjing Agricultural University (Nanjing, China). The animal experiment was undertaken following the guidelines of the Animal Ethics Committee of Nanjing Agricultural University, China. During a surgical procedure that required only a short post-operative recovery time, a small cannula constructed from polyvinylchloride (PVC) was inserted into the rumen of each goat. The surgical recovery time was two weeks. During the next 2 wks of adaptation periods, goats were offered free access to the same diet containing a forage-to-concentrate ratio (F∶C) of 65∶35. After adaptation, goats were randomly allocated to two groups. One group was fed a control diet comprising 65% forage and 35% mixed concentrate (low concentrate group, LC), while the other group received a high-grain diet containing 43% corn, 5% wheat bran, 17% mixed concentrate and 35% forage (high concentrate group, HC). Both groups of goats were fed daily at 8:00 and 18:00, and feed was always provided in an amount that met or exceeded the animal's nutritional requirements. The animals were fed the respective diets for 6 wks, and had free access to water during the experimental period.

### Rumen sampling and analysis

Fifteen minutes prior to feed delivery and 2, 4, 6 and 8 h after feed delivery on 2 consecutive days during wk 2 and 6, ten milliliter rumen fluids was collected with a nylon bag and the pH value was measured immediately with pH-meter.

The rumen fluid was collected and each sample was transferred into a 50-mL sterile tube and kept on ice until transported to the laboratory for the initial processing before LPS determination as described by Gozho et al. [29]. Briefly, rumen fluid samples were centrifuged at 10,000× g for 45 min at 4°C and the supernatant was aspirated gently to prevent its mixing with the pellet and passed through a disposable 0.22-µm LPS-free filter. The filtrate was collected in a sterile glass tube (previously heated at 180°C for 4 h) and heated at 100°C for 30 min. Samples were cooled at room temperature (25°C) for 10 min and stored at −20°C for LPS analysis.

The concentration of LPS in rumen fluid was measured by a Chromogenic End-point Tachypleus Amebocyte Lysate Assay Kit (Chinese Horseshoe Crab Reagent Manufactory Co. Ltd, Xiamen, China). Pretreated rumen fluid samples were diluted until their LPS concentrations were in the range of 0.1 to 1 endotoxin units (EU)/mL relative to the reference endotoxin, and assayed as described by Gozho *et al.* [29].

### Blood sampling and analysis

For consecutive collection the blood samples, each goat was installed a jugular catheter with a minor surgical operation with local anesthesia at 3 days prior to sampling. Fifteen minutes before feed delivery and at 15, 30, 60, 90,120 and 180 min after feed delivery at the end of wk 6, blood samples were collected from jugular vein into 10 mL anti-coagulant tubes and centrifuged at 3,500×g at 4°C for 15 min for collection of plasma. Plasma was stored at −20°C for analysis.

The concentration of LPS in plasma was determined by a Chromogenic End-point Tachypleus Amebocyte Lysate Assay Kit (Chinese Horseshoe Crab Reagent Manufactory Co. Ltd, Xiamen, China). The ranges of assay sensitivity were from 0.1 to 1.0 EU/mL.

### Transmission electron microscopy of TJ

Colon and cecum tissue samples were separated and fixed immediately with 2% glutaraldehyde, post-fixed with 1% osmium tetroxide, and embedded in resin. Ultrathin sections were cut and stained with uranyl acetate and lead citrate. Epithelial tissues ultrastructure was determined with a transmission electron microscope (Hitachi H-7650, Hitachi Technologies, Tokyo, Japan).

### Histopathology

Specimens of the intestinal wall of the colon and cecum were prepared for histological examination by fixing in 4% formaldehyde-buffered solution, embedding in paraffin, and sectioning. Histological damage scoring was determined with criteria adapted from Wu et al. [48]. Briefly, the damage score consisted of a score for the severity of epithelial injury (graded 0–3, from absent to mild including superficial epithelial injury, moderate including focal erosions, and severe including multifocal erosions), the extent of inflammatory cell infiltrate (graded 0–3, from absent to transmural), and goblet cell depletion (0–1). In each case a numerical score was assigned. Three tissue sections from each animal were coded and examined by Dr. Xiangzhen Shen and Dr. Hongli Wang to prevent observer bias.

### RNA isolation, cDNA synthesis and real-time PCR

Colon and cecum mucosa tissues were quickly collected and immediately frozen in liquid nitrogen, and stored at −80°C until RNA isolation. Total RNA was extracted from colon and cecum samples with Trizol Reagent (15596026, Invitrogen). Concentration and quality of the RNA were measured by NanoDrop ND-1000 Spectrophotometer (Thermo, USA). Then two micrograms of total RNA were treated with RNase-Free DNase (M6101, Promega, USA) and reverse-transcribed according to manufacturer's instructions. Real-time PCR was performed in Mx3000P (Stratagene, USA). β-actin, which is not affected by the experimental factors, was chosen as the reference gene. In order to control PCR efficiency, two microliters of diluted cDNA (1∶40, vol/vol) was used in each real-time PCR. The following PCR protocols were initial denaturation (1 min at 95°C), then a three-step amplification program (20 s at 95°C, 20–30 s at 62°C, 30 s at 72°C) was repeated 45 times. Primers were synthesized by Generay Company (Shanghai, China), and the information was shown in Table 1. The method of 2^−ΔΔCt^ was used to analyze the real-time PCR results and gene mRNA levels were expressed as the fold change relative to the mean value of control group.

**Table 1:** PCR primer sequences of the target genes.

Target genes	Reference/Genbank accession	PCR products (bp)	Primer sequences
β-actin	AF_481159	260	F: 5′-CGGGATCCATCCTGCGTCTGGACCTG -3′
			R: 5′-GGAATTCGGAAGGAAGGCTGGAAGAG -3′
TLR4	JQ342090.1	195	F: 5′-GTTTCCACAAGAGCCGTAA-3′
			R: 5′-TGTTCAGAAGGCGATAGAGT-3′
MyD88	JQ308783.1	98	F: 5′-ACAAGCCAATGAAGAAAGAG-3′
			R: 5′-GAGGCGAGTCCAGAACC -3′
TNF-α	AF276985.1	173	F: 5′-CAAGTAACAAGCCGGTAGCCC-3′
			R: 5′-CCTGAAGAGGACCTGCGAGTAG-3′
IL-1β	D63351.1	172	F: 5′-GAAGAGCTGCACCCAACA-3′
			R: 5′-CAGGTCATCATCACGGAAG-3′
Caspase-3	AF068837.1	98	F: 5′-GGTTCATCCAGGCTCTTT-3′
			R: 5′-TTCTGTCGCTACCTTTCG-3′
Caspase-8	NM_001045970	149	F: 5′-GGCTCCTCTGAGATGCTG-3′
			R: 5′-TGCTCCCGTGCTATGCTA-3′
TLR2	NM_174197.2	141	F: 5′-ACTTCTCCCATTTCCG-3′
			R: 5′-GCCACTCCAGGTAGGT-3′

TLR4, Toll-like receptor 4; MyD88, myeloid differentiating factor 88; TNF-α, tumor necrosis factor alpha; IL-1β, interleukin-1 beta; TLR2, Toll-like receptor 2.

### Caspase-3 and -8 activity analysis

100 mg frozen colon and cecum tissues were minced and homogenized in 1 mL of ice-cold cytoplasm RIPA containing the protease inhibitor cocktail Complete EDTA-free (Roche, Penz-berg, Germany). The homogenates were centrifuged at 12,000 rpm for 15 min at 4°C and then collected the supernatant. Protein concentration was determined using a BCA Protein Assay kit (Pierce, Rockford, IL, USA), and then diluted to the same concentration and stored at −80°C for subsequent caspase enzyme activity analysis. Caspase-3 and caspase-8 enzyme activity of hindgut tissues were measured by caspase activity Assay Kits (G007 and G008, respectively, Jiancheng Bioengineering Institute, Nanjing, China). The procedures were performed according to the manufacture's instruction.

### Western blotting

Frozen colon and cecum mucosa tissues (100 µg) were minced and homogenized in 1 mL of ice-cold homogenization buffer RIPA containing the protease inhibitor cocktail Complete EDTA-free (Roche, Penz-berg, Germany). The homogenates were centrifuged at 12,000 rpm for 20 min at 4°C and then collected the supernatant fraction. Protein concentration was determined using a BCA Protein Assay kit (Pierce, Rockford, IL, USA). Eighty micrograms of protein extract from each sample was then loaded onto 7.5% and 15% SDS-PAGE gels and the separated proteins were transferred onto the nitrocellulose membranes (Bio Trace, Pall Co, USA). After transferred, membranes were blocked for 2 h at room temperature in blocking buffer and then membranes were incubated with the following primary antibodies: NF-κB p65 (1∶500; sc-372, Santa Cruz) and GAPDH (1∶10000; AP0066, Bioworld, USA) in dilution buffer overnight at 4°C. After several washes in Tris-Buffered-Saline with Tween (TBST), membranes were incubated with goat anti-rabbit horseradish peroxidase (HRP)-conjugated secondary antibodies (1∶10000; Bioworld, USA) in dilution buffer for 2 h at room temperature. Finally, the blot was washed and signal was detected by enhanced chemiluminescence (ECL) using the LumiGlo substrate (Super Signal West Pico, Pierce, USA), and the signals were recorded by an imaging System (Bio-Rad, USA), and analyzed with Quantity One software (Bio-Rad, USA).

### Statistical analysis

The results were expressed as mean ± SEM. The data of ruminal pH and LPS in plasma and ruminal fluid were analyzed for differences due to diet, feeding time, and their interactions by Univariate using the General Linear Models of SPSS 11.0 for Windows (StatSoft Inc, Tulsa, OK, USA). The differences in caspase activities, mRNA and protein expression between two groups were analyzed by using the post hoc analysis with the least significant difference test following ANOVA of SPSS 11.0. The differences of the histological scoring between two groups were analyzed by using non-parametric statistical analysis (Wilcoxon rank sum test). Data was considered statistically significant when *P*<0.05. Numbers of replicates used for statistics are noted in the Tables and Figures.

## References

[1] Gaebel G, Bell M, Martens H (1989) The effect of low mucosal pH on sodium and chloride movement across the isolated rumen mucosa of sheep. Q J Exp Physiol 74: 35–44. 2717703 10.1113/expphysiol.1989.sp003237

[2] Andersen PH, Hesselholt M, Jarlov N (1994) Endotoxin and Arachidonic-Acid Metabolites in Portal, Hepatic and Arterial Blood of Cattle with Acute Ruminal Acidosis. Acta Vet Scand 35: 223–234. 7847191 10.1186/BF03548327 PMC8101378

[3] Khafipour E, Krause DO, Plaizier JC (2009) A grain-based subacute ruminal acidosis challenge causes translocation of lipopolysaccharide and triggers inflammation. J Dairy Sci 92: 1060–1070. 19233799 10.3168/jds.2008-1389

[4] Zebeli Q, Ametaj BN (2009) Relationships between rumen lipopolysaccharide and mediators of inflammatory response with milk fat production and efficiency in dairy cows. J Dairy Sci 92: 3800–3809. 19620662 10.3168/jds.2009-2178

[5] Chin AC, Flynn AN, Fedwick JP, Buret AG (2006) The role of caspase-3 in lipopolysaccharide-mediated disruption of intestinal epithelial tight junctions. Can J Physiol Pharm 84: 1043–1050. 10.1139/y06-056 17218970

[6] Steele MA, Croom J, Kahler M, AlZahal O, Hook SE, et al (2011) Bovine rumen epithelium undergoes rapid structural adaptations during grain-induced subacute ruminal acidosis. Am J Physiol-Reg I 300: 1515–1523. 10.1152/ajpregu.00120.2010 21451145

[7] Plaizier JC, Khafipour E, Li S, Gozho GN, Krause DO (2012) Subacute ruminal acidosis (SARA), endotoxins and health consequences. Anim Feed Sci Tech 172: 9–21.

[8] Khafipour E, Krause DO, Plaizier JC (2009) Alfalfa pellet-induced subacute ruminal acidosis in dairy cows increases bacterial endotoxin in the rumen without causing inflammation. J Dairy Sci 92: 1712–1724. 19307653 10.3168/jds.2008-1656

[9] Li S, Khafipour E, Krause DO, Kroeker A, Rodriguez-Lecompte Jc, et al (2012) Effects of subacute ruminal acidosis challenges on fermentation and endotoxins in the rumen and hindgut of dairy cows. J Dairy Sci 95: 294–303. 22192209 10.3168/jds.2011-4447

[10] Penner GB, Steele MA, Aschenbach JR, McBride BW (2011) Ruminant Nutrition Symposium: Molecular adaptation of ruminal epithelia to highly fermentable diets. J Anim Sci 89: 1108–1119. 20971890 10.2527/jas.2010-3378

[11] Gassler N, Rohr C, Schneider A, Kartenbeck J, Bach A, et al (2001) Inflammatory bowel disease is associated with changes of enterocytic junctions. Am J Physiol-Gastr L 281: 216–228. 10.1152/ajpgi.2001.281.1.G216 11408275

[12] Zeissig S, Burgel N, Gunzel D, Richter J, Mankertz J, et al (2007) Changes in expression and distribution of claudin 2, 5 and 8 lead to discontinuous tight junctions and barrier dysfunction in active Crohn's disease. Gut 56: 61–72. 16822808 10.1136/gut.2006.094375 PMC1856677

[13] John LJ, Fromm M, Schulzke JD (2011) Epithelial Barriers in Intestinal Inflammation. Antioxid Redox Sign 15: 1255–1270. 10.1089/ars.2011.3892 21294654

[14] Turner JR (2009) Intestinal mucosal barrier function in health and disease. Nat Rev Immunol 9: 799–809. 19855405 10.1038/nri2653

[15] Furrie E, Macfarlane S, Thomson G, Macfarlane GT (2005) Toll-like receptors-2,-3 and-4 expression patterns on human colon and their regulation by mucosal-associated bacteria. Immunology 115: 565–574. 16011525 10.1111/j.1365-2567.2005.02200.x PMC1782176

[16] Akira S (2009) Pathogen recognition by innate immunity and its signaling. P Jpn Acad B-Phys 85: 216–216. 10.2183/pjab.85.143 PMC3524297 19367086

[17] Neish AS (2009) Microbes in Gastrointestinal Health and Disease. Gastroenterology 136: 65–80. 19026645 10.1053/j.gastro.2008.10.080 PMC2892787

[18] Sharma R, Young C, Neu J (2010) Molecular Modulation of Intestinal Epithelial Barrier: Contribution of Microbiota. J Biomed Biotechnol 2010: 1–15. 10.1155/2010/305879 PMC2817557 20150966

[19] Poltorak A, He XL, Smirnova I, Liu MY, Van Huffel C, et al (1998) Defective LPS signaling in C3H/HeJ and C57BL/10ScCr mice: Mutations in Tlr4 gene. Science 282: 2085–2088. 9851930 10.1126/science.282.5396.2085

[20] Takeda K, Kaisho T, Akira S (2003) Toll-like receptors. Annu Rev Immunol 21: 335–376. 12524386 10.1146/annurev.immunol.21.120601.141126

[21] Baldwin AS Jr (1996) The NF-kappa B and I kappa B proteins: new discoveries and insights. Annu Rev Immunol 14: 649–683. 8717528 10.1146/annurev.immunol.14.1.649

[22] Williams JM, Duckworth CA, Watson AJ, Frey MR, Miguel JC, et al (2013) A mouse model of pathological small intestinal epithelial cell apoptosis and shedding induced by systemic administration of lipopolysaccharide. Dis Mod Mech 6: 1388–1399. 10.1242/dmm.013284 PMC3820262 24046352

[23] Liu JH, Xu TT, Liu YJ, Zhu WY, Mao SY (2013) A high-grain diet causes massive disruption of ruminal epithelial tight junctions in goats. Am J Physiol-Reg I 305: 232–241. 10.1152/ajpregu.00068.2013 23739344

[24] Klevenhusen F, Hollmann M, Podstatzky-Lichtenstein L, Krametter-Frotscher R, et al (2013) Feeding barley grain-rich diets altered electrophysiological roperties and permeability of the ruminal wall in a goat model. J Dairy Sci 96: 2293–2302. 23403198 10.3168/jds.2012-6187

[25] Zebeli Q, Metzler-Zebeli BU (2012) Interplay between rumen digestive disorders and diet-induced inflammation in dairy cattle. Research in Veterinary Science 93: 1099–1108. 22370295 10.1016/j.rvsc.2012.02.004

[26] Hollmann M, Miller I, Hummel K, Sabitzer S, Metzler-Zebeli BU, et al (2013) Downregulation of Cellular Protective Factors of Rumen Epithelium in Goats Fed High Energy Diet. Plos One 8: e81602. 24349094 10.1371/journal.pone.0081602 PMC3857193

[27] Emmanuel DGV, Madsen KL, Churchill TA, Dunn SM, Ametaj BN (2007) Acidosis and lipopolysaccharide from Escherichia coli B: 055 cause hyperpermeability of rumen and colon tissues. J Dairy Sci 90: 5552–5557. 18024746 10.3168/jds.2007-0257

[28] Elam CJ (1976) Acidosis in feedlot cattle: practical observations. J Anim Sci 43: 898–901. 977503 10.2527/jas1976.434898x

[29] Gozho GN, Plaizier JC, Krause DO, Kennedy AD, Wittenberg KM (2005) Subacute ruminal acidosis induces ruminal lipopolysaccharide endotoxin release and triggers an inflammatory response. J Dairy Sci 88: 1399–1403. 15778308 10.3168/jds.S0022-0302(05)72807-1

[30] Kleen JL, Hooijer GA, Rehage J, Noordhuizen JPTM (2003) Subacute ruminal acidosis (SARA): a review. J Vet Med A 50: 406–414. 10.1046/j.1439-0442.2003.00569.x 14633219

[31] Krause KM, Oetzel GR (2005) Inducing subacute ruminal acidosis in lactating dairy cows. J Dairy Sci 88: 3633–3639. 16162537 10.3168/jds.S0022-0302(05)73048-4

[32] Plaizier JC, Krause DO, Gozho GN, McBride BW (2008) Subacute ruminal acidosis in dairy cows: The physiological causes, incidence and consequences. Vet J 176: 21–31. 18329918 10.1016/j.tvjl.2007.12.016

[33] Metzler-Zebeli BU, Hollmann M, Sabitzer S, Podstatzky-Lichtenstein L, Klein D, et al (2013) Epithelial response to high-grain diets involves alteration in nutrient transporters and Na+/K+-ATPase mRNA expression in rumen and colon of goats. J Anim Sci 91: 4256–4266. 23825322 10.2527/jas.2012-5570

[34] Motoi Y, Oohashi T, Hirose H, Hiramatsu M, Miyazaki S, et al (1993) Turbidimetric-Kinetic Assay of Endotoxin in Rumen Fluid or Serum of Cattle Fed Rations Containing Various Levels of Rolled Barley. J Vet Med Sci 55: 19–25. 8461422 10.1292/jvms.55.19

[35] Emmanuel DGV, Dunn SM, Ametaj BN (2008) Feeding high proportions of barley grain stimulates an inflammatory response in dairy cows. J Dairy Sci 91: 606–614. 18218747 10.3168/jds.2007-0256

[36] Metzler-Zebeli BU, Schmitz-Esser S, Klevenhusen F, Podstatzky-Lichtenstein L, Wagner M, et al (2013) Grain-rich diets differently alter ruminal and colonic abundance of microbial populations and lipopolysaccharide in goats. Anaerobe 20: 65–73. 23474085 10.1016/j.anaerobe.2013.02.005

[37] Satoh M, Ando S, Shinoda T, Yamazaki M (2008) Clearance of bacterial lipopolysaccharides and lipid A by the liver and the role of argininosuccinate synthase. Innate Immun-London 14: 51–60. 10.1177/1753425907087267 18387919

[38] Graham C, Simmons NL (2005) Functional organization of the bovine rumen epithelium. Am J Physiol-Reg I 288: 173–181. 10.1152/ajpregu.00425.2004 15319221

[39] Nusrat A, Turner JR, Madara JL (2000) Molecular physiology and pathophysiology of tight junctions IV. Regulation of tight junctions by extracellular stimuli: nutrients, cytokines, and immune cells. Am J Physiol-Gastr L 279: 851–857. 10.1152/ajpgi.2000.279.5.G851 11052980

[40] Wainwright MS, Rossi J, Schavocky J, Crawford S, Steinhorn D, et al (2003) Protein kinase involved in lung injury susceptibility: Evidence from enzyme isoform genetic knockout and in vivo inhibitor treatment. P Natl Acad Sci USA 100: 6233–6238. 10.1073/pnas.1031595100 PMC156355 12730364

[41] MacCallum A, Hardy SP, Everest PH (2005) Campylobacter jejuni inhibits the absorptive transport functions of Caco-2 cells and disrupts cellular tight junctions. Microbiology 151: 2451–2458. 16000735 10.1099/mic.0.27950-0

[42] Lan A, Lagadic-Gossmann D, Lemaire C, Brenner C, Jan G (2007) Acidic extracellular pH shifts colorectal cancer cell death from apoptosis to necrosis upon exposure to propionate and acetate, major end-products of the human probiotic propionibacteria. Apoptosis 12: 573–591. 17195096 10.1007/s10495-006-0010-3

[43] Caradonna L, Amati L, Magrone T, Pellegrino M, Jirillo E, et al (2000) Enteric bacteria, lipopolysaccharides and related cytokines in inflammatory bowel disease: biological and clinical significance. J Endotoxin Res 6: 205–214. 11052175

[44] Liu X, Wang JM (2011) Iridoid glycosides fraction of Folium syringae leaves modulates NF-κB signal pathway and intestinal epithelial cells apoptosis in experimental colitis. PLoS One 6: e24740. 21931839 10.1371/journal.pone.0024740 PMC3172289

[45] Neurath MF, Pettersson S, Meyer zum Büschenfelde KH, Strober W (1996) Local administration of antisense phosphorothioate oligonucleotides to the p65 subunit of NF-kappa B abrogates established experimental colitis in mice. Nat Med 9: 998–1004. 10.1038/nm0996-998 8782457

[46] Yue C, Ma BQ, Zhao YZ, Li QR, Li JS (2012) Lipopolysaccharide-Induced Bacterial Translocation Is Intestine Site-Specific and Associates with Intestinal Mucosal Inflammation. Inflammation 35: 1880–1888. 22821406 10.1007/s10753-012-9510-1

[47] Beeman N, Webb PG, Baumgartner HK (2012) Occludin is required for apoptosis when claudin-claudin interactions are disrupted. Cell Death Dis 23: e273. 10.1038/cddis.2012.14 PMC3288343 22361748

[48] Wu X, Vallance BA, Boyer L, Bergstrom KS, Walker J, et al (2008) Saccharomyces boulardii ameliorates Citrobacter rodentium-induced colitis through actions on bacterial virulence factors. Am J Physiol Gastrointest Liver Physiol 294: G295–306. 18032474 10.1152/ajpgi.00173.2007

